# Wawruch, on the Causes and Symptoms of Tape-Worm

**Published:** 1851-07

**Authors:** 


*Wawruch, on the Causes and Symptoms of Tape-Worm.*—The following is an extract from an abstract, contained in the *Gazette Medicale* for 2d October, 1841, of a paper published by Prof. Wawruch in the *Medicinische Jahrbucher der Œsterreichischen Staaten.*

During the period of twenty years, Dr. Wawruch treated 206 persons affected with tænia; 71 men, and 135 women. The oldest person was aged fifty-four; the youngest, three and a half years. Twenty-two were under fifteen years of age; and among these were six girls who had not menstruated. Most of the patients were from fifteen to forty years old. The patients belonged to the middle and lower classes; they nearly all inhabited the district lying along the Danube, or lived in low and damp dwellings. They followed very different occupations, and their mode of life was various. It is remarkable that the same conditions which give rise to scurvy in Vienna also produce tape-worm. The articles of food which seemed chiefly to engender tape-worm were, bad bread, meals of milk, butter, cheese, potatoes, pork and mutton, and bad water. The diseases which preceded the formation of tænia were gastric and cutaneous affections, but especially gastric and intermittent fevers. There were observed forty-three cases of intermittent fever, twenty of gastric fever, sixteen of typhoid fever, ten of ringworm and herpes, forty-two of itch, eight of scarlatina, thirteen of measles, and two of chronic urticaria. Scurvy, syphilis, chlorosis, and other diseases which affect digestion, were also observed to precede tænia; but, in general, few of the individuals affected with tape-worm had not had lumbrici when young. The influence of hereditary predisposition is very doubtful. Dr. Wawruch only saw two instances; in one, a mother and daughter, in another, a father and son, had tænia. Irregularity of the menstrual function seemed common; thus, thirteen females had only menstruated at the age of 16 years, twelve at 17, nine at 18, seven at 19, and one at 20. The duration of the disease was from a few months to ten, twelve, fifteen, twenty, twenty-five, and, in one case, even to thirty-five years. Of the 206 patients, three only, who were foreigners, had *bothriocephalus;* the others had *tænia solium.*

*Symptoms.* The symptoms of tænia are very various; the following are stated by Dr. Wawruch to be the most constant.

1. Dull pain in the frontal region, vertigo, noises in the ears; and in some, a kind of idiosyncrasy for music. 2. Eyes dull, surrounded by a blue ring; œdema of the upper eyelid; pupils dilated; frequent involuntary action of the muscles of the eye; various aberrations of sight, as amblyopia, diplopia, ormuscæe volitantes. One patient complained of a periodical blindness during the day. 3. Frequent change in the color of the face; lips lead-colored; a peculiar look about the mouth and nose; the appearance of general cachexia is not constant, for the patients often preserve a flourishing aspect for some time: many of them say they have become thinner. 4. Anorexia, alternating with such a voracious appetite that the patients faint if they are not supplied quickly enough with food; desire for certain articles, as carrots, milk, wine, bread, etc. 5. Fetid breath, clayey taste, loaded tongue, salivation, nausea, and even vomiting of liquid, as clear as water, in the morning. 6. Itching of the nose and anus, and of the vagina in females; grinding of the teeth, especially during sleep; accumulation of a clear fluid in the mouth; transient heat, languor, palpitation. The sensation of a cold hand pressing on the heart (considered as a pathognomic symptom by Reinlein) has never been observed. 7. Abdomen tumefied; borborygmi; a sensation of suction, constriction, and pricking around the umbilicus, and undulatory movements, as of a foreign body in the intestines, especially in the morning; cessation of these symptoms after taking farinaceous food, hot bread, or *cafe au lait;* frequently diarrhœa, alternating with constipation. 8. When the disease has continued for some time, and the patients are of an irritable temperament, there often arise a train of nervous symptoms, as melancholia, syncope, aberrations of the senses, disturbed sleep, frightful dreams, partial and general convulsions, chorea, epilepsy, aphonia, and loss of speech. 9. The most certain pathognomonic symptom is the evacuation of one or more portions of tape-worm, either spontaneously, or after certain severe illnesses—as scarlatina, typhoid fever, pneumonia, etc.; or after the use of certain articles of food, as garlic, horseradish, cucumber, etc.; or after the administration of anthelmintic medicines. The portions are sometimes evacuated at indeterminate periods, but generally at new moon, or at the decrease of the moon; and, at these times, also, the other symptoms are increased in severity.—*London Jour. Med.*

